# Anxiety and autistic traits in adults: a systematic review and meta-analysis

**DOI:** 10.3389/fpsyg.2025.1680267

**Published:** 2025-10-29

**Authors:** Lucía Torices Callejo, Laura Herrero, Miguel Ángel Pérez Nieto

**Affiliations:** ^1^HM Faculty of Health Sciences, Universidad Camilo José Cela, Madrid, Spain; ^2^HM Hospitals Health Research Institute, Madrid, Spain

**Keywords:** autism, autistic traits, anxiety, systematic review, meta-analysis

## Abstract

**Background:**

Autistic traits are distributed along a continuum, and some individuals exhibit subclinical characteristics without meeting diagnostic criteria for autism spectrum disorder (ASD). This population, referred to as the broader autism phenotype (BAP), has been associated with increased prevalence of anxiety symptoms. Given that these individuals often do not access clinical services or receive interventions, understanding how anxiety manifests within this group is essential for improving psychological well-being and quality of life. Although research on autism and psychopathology has expanded in recent years, few studies have explored this relationship in adults with BAP from a dimensional and transdiagnostic perspective.

**Objective:**

This study aimed to systematically review and synthesize recent empirical evidence on the relationship between autistic traits and anxiety symptoms in adults, and to assess whether this association is statistically significant.

**Methods:**

A systematic search was conducted across four databases (PubMed, Web of Science, Scopus, Dialnet) for peer-reviewed articles published between 2013 and 2023. Studies were included if they used validated instruments to assess autistic traits (e.g., AQ, ADOS-2) and anxiety (e.g., HADS, STAI, GAD-7, BAI). A total of 18 independent samples from 13 studies were included. Effect sizes (Hedges’ g) were calculated and synthesized using a random-effects model. Heterogeneity and publication bias were also examined.

**Results:**

Findings were mixed: 55% of the included studies reported positive effect sizes and 45% negative. However, the overall effect size was not statistically significant (*g* = 0.0234, SE = 0.235, 95% CI: −0.438 to 0.483, *p* = 0.921), with substantial heterogeneity across studies (*I*^2^ = 99.83%). Larger studies tended to report positive associations, while smaller studies yielded negative or inconsistent effects. Inconsistencies in measurement tools, particularly across AQ versions, contributed to this variability.

**Conclusion:**

Although a significant association was not confirmed, the high heterogeneity highlights the need for more standardized approaches to evaluating autistic traits in non-clinical adult populations. These findings underscore the complexity of subclinical autism and support the relevance of transdiagnostic research frameworks to better understand its relationship with anxiety.

## 1 Introduction

Autism is a set of characteristics and behaviors observed in varying degrees across the population. This dimensional understanding of autism, in which traits are distributed continuously in the general population rather than confined to clinical diagnoses, has gained increasing empirical support in recent years (Barros et al., 2022). Individuals with qualitatively similar traits to ASD but not meeting all diagnostic criteria are referred to as subclinical autistics or the broad autism phenotype (BAP) (Ruzich et al., 2015; Wheelwright et al., 2010; Godoy-Gimenez et al., 2018). Recent research has emphasized the importance of BAP characteristics in understanding vulnerability to emotional difficulties, including mood and anxiety disorders, particularly in young adults (Kurtz et al., 2023). In this study, we differentiate three levels of autistic expression: (1) clinically diagnosed Autism Spectrum Disorder (ASD), (2) individuals exhibiting subclinical features falling within the Broad Autism Phenotype (BAP), and (3) autistic traits measured dimensionally in the general population. While these categories are conceptually distinct, prior research has sometimes conflated them, limiting the clarity of findings and theoretical interpretations. Addressing this differentiation is critical for accurately assessing the relationship between autistic traits and anxiety in non-clinical populations. In particular, overlooking the distinction between clinical autism diagnoses and subclinical traits may obscure unique pathways through which autistic characteristics influence emotional regulation and anxiety in the general population (Normansell-Mossa et al., 2021).

Research on autism is extensive, although it is not as comprehensive when it comes specifically to the adult population. This gap is especially evident in studies examining subclinical autistic traits in neurotypical adult samples, where the representation remains limited despite growing interest in dimensional approaches (Barros et al., 2022). Furthermore, it is fraught with challenges, particularly when investigating psychological or mental health aspects. For instance, symptoms of anxiety may overlap with core autistic traits such as social withdrawal or cognitive rigidity, making it difficult to disentangle comorbid conditions, especially when relying solely on self-report instruments (Ni et al., 2023). The assessment instruments employed lack specificity, autism symptoms are commonly confused with anxiety indicators, masking tendencies are often overlooked, among other issues. Moreover, masking behaviors may lead to underestimation of emotional distress in individuals with subclinical autistic traits, as they may camouflage symptoms in socially demanding contexts (Normansell-Mossa et al., 2021). Most studies on autism in adults use samples of family members of individuals diagnosed with ASD but lack representation from the general population. These investigations predominantly focus on social processes (Morrison et al., 2018), indicating that individuals with BAP features, as well as individuals displaying milder forms of ASD, exhibit reduced interpersonal abilities due to deficits in social cognition and social skills (Sasson et al., 2013) and higher levels of unwanted loneliness (Jobe and White, 2007).

The adult population with autism spectrum characteristics—including both formally diagnosed individuals and those with subclinical traits—faces an increased risk of co-occurring mental health conditions mental health conditions, Attention deficit hyperactivity disorder (ADHD) stands out as the predominant diagnosis in adults with autism spectrum disorder (ASD), while less common are those associated with substance use (Lugo-Marin et al., 2019; Hollocks et al., 2019). Some studies report a 9.1% prevalence of personality disorders in individuals diagnosed with ASD (Lugo-Marin et al., 2019). Some traits, such as limited emotional expressiveness or a preference for solitary activities, may superficially resemble features of certain personality disorders, such as schizoid personality disorder. However, this overlap is partial and does not account for differences in motivational and cognitive underpinnings. In this context, anxiety has emerged as one of the most prevalent and impairing co-occurring conditions—not only in individuals with ASD, but also in those exhibiting elevated autistic traits without a formal diagnosis (Joshi et al., 2013; Barros et al., 2022).

Anxiety, as defined by Clark and Beck (2012), is a complex response system involving behavioral, physiological, affective, and cognitive aspects. This multidimensional construct is particularly relevant when investigating autistic traits, as individuals with such traits often report heightened anticipatory responses to uncertainty and social evaluation. The system is activated in the anticipation of events perceived as highly aversive—unpredictable, uncontrollable, and potentially threatening to vital interests. In other words, it is the anticipation of a future threat (American Psychiatric Association, 2013). This response shifts from being adaptive to pathological when it becomes excessive or persists beyond standard developmental stages. Such dysregulation may be especially common in individuals with elevated autistic traits, where intolerance of uncertainty and altered sensory processing have been proposed as key mechanisms (Normansell-Mossa et al., 2021).

The role of anxiety within the autism condition has evolved over time. Historical perspectives mentioned anxiety to explain core autism symptoms. For instance, restricted behaviors and interests were defined as anxiety self-regulation strategies (Despert, 1965) triggered by difficulties in environmental understanding (Howlin, 1998). In addition, rigidity in autism was explained as a coping mechanism for constant uncertainty (Schopler and Mesibov, 1994), and the desire for environmental invariance as a response to anxiety in social contexts (Kanner, 1944). However, this view has been controversial, fueled by all the cases where anxiety is absent in the autistic context (Wing et al., 1977). According to Paula (2016), some prototypically autistic behaviors intensify when interacting with anxiety. For example, this includes heightened insistence on routines (rigidity) or the accentuation of socially inappropriate behaviors. Contemporary models build upon these early hypotheses by suggesting that traits such as intolerance of uncertainty or emotional dysregulation may mediate the link between autism and anxiety across the spectrum, including in individuals without a formal diagnosis (Barros et al., 2022; Normansell-Mossa et al., 2021).

Despite the high prevalence of anxiety in individuals with diagnosed autism, research on its presence and mechanisms in individuals with subclinical autistic traits—such as those within the broader autism phenotype (BAP)—remains limited. Moreover, studies diverge considerably in terms of inclusion criteria, sample composition, and psychometric rigor of the instruments used. These inconsistencies complicate the synthesis of findings and point to a need for greater conceptual clarity and methodological coherence in the field. This underrepresentation limits the generalizability of findings and hinders the development of interventions targeting populations beyond clinical settings.

Estimates of anxiety prevalence in adults with ASD vary widely depending on diagnostic methods and sample characteristics. While some studies report rates as high as 70% (Charlot et al., 2008; Mazefsky et al., 2008), others suggest more conservative figures around 27% (Paula, 2016), or even lower when relying on clinical records alone (Tsakanikos et al., 2011). Anxiety manifestations in autistic and non-autistic individuals are generally not linked to the same psychosocial variables. However, exceptions arise in cases where anxiety co-occurs with ASD (Paula, 2013, 2016). Such variability underscores the need for standardized measurement approaches and inclusion of non-clinical samples.

The prevalence of clinically significant anxiety is notably high in the autistic population, though not universal. Consequently, some experts posit that anxiety may constitute an element in the diathesis of the autistic condition. In light of this, individuals with autism are predisposed to anxiety. Wood and Gadow (2010) propose anxiety as a factor consolidating subtypes of ASD rather than a mere comorbidity. This perspective aligns with transdiagnostic models that conceptualize anxiety as a shared vulnerability factor modulated by neurodevelopmental traits (Van Steensel et al., 2011; Wood and Gadow, 2010).

This information prompts consideration of anxiety co-occurring with autism, emphasizing the importance of understanding associated challenges. This correlation is linked to lower quality of life (Adams et al., 2020; Lin and Huang, 2019), heightened anger (Townsend et al., 2022), and decreased community participation (Ambrose et al., 2022). Understanding this interaction is essential not only for accurate diagnosis but also for tailoring interventions aimed at improving emotional well-being and adaptive functioning in adults with autistic traits.

Despite growing interest in the relationship between autistic traits and anxiety, the existing literature remains fragmented, primarily due to methodological heterogeneity and a predominant focus on clinical samples. Studies diverge significantly in the instruments used to assess both constructs, which limits comparability and hampers theoretical integration. Previous systematic reviews have largely centered on individuals with formal ASD diagnoses (Hollocks et al., 2013; Lugo-Marin et al., 2019; Jenkinson et al., 2020), and have often examined broader domains such as executive functioning (Demetriou et al., 2018; Velikonja et al., 2019) or general psychopathology prevalence (Park et al., 2019; Hollocks et al., 2019). In contrast, the present study focuses specifically on adults from the general population and aims to explore how autistic traits relate to anxiety symptoms through a meta-analytic synthesis. Furthermore, it evaluates how the selection of measurement instruments influences this association, thereby addressing a key gap in the literature and contributing to a more refined understanding of autism-related anxiety from a dimensional and transdiagnostic perspective.

## 2 Methods

### 2.1 Literature search and study identification

#### 2.1.1 Information sources and search strategy

The review followed the PRISMA 2020 guidelines (Liberati et al., 2009;Page et al., 2021) for systematic reviews and meta-analyses. Given the wide range of approaches, disciplines, and instruments used to assess both autism and anxiety, a search strategy based on the measurement tools for both variables was adopted. This decision aimed to reduce methodological heterogeneity and ensure a minimum level of consistency among the selected studies. The instruments were chosen based on their frequent use in scientific literature, as well as their sound psychometric properties in adult populations, including construct validity and internal reliability.

A systematic search was conducted across four electronic databases: Web of Science (WOS), Scopus, PubMed, and Dialnet. The search was restricted to peer-reviewed journal articles published between January 2013 and December 2023. The search strategy used Boolean operators and keyword combinations, as outlined in Table 1: Autism Spectrum Quotient (AQ) (Baron-Cohen et al., 2001), Autism Diagnostic Observation Schedule–2 (ADOS-2) (Lord et al., 2012), Hospital Anxiety and Depression Scale (HADS) (Spitzer et al., 2015; Zigmond et al., 1993), Generalized Anxiety Disorder 7 (GAD-7), State–Trait Anxiety Inventory (STAI) (Spielberger et al., 1999), Beck Anxiety Inventory (BAI) (Beck et al., 1988), and Symptom Checklist-90-Revised (SCL-90-R) (Derogatis et al., 1977).

**Table 1:** Search strategy.

No	Search
#1	Autism* AND anxie* NOT child*
#2	AQ* AND (ADOS2 OR ADOS-2) AND HADS AND STAI AND GAD* AND SCL* AND BAI NOT child*

**Figure 1:**
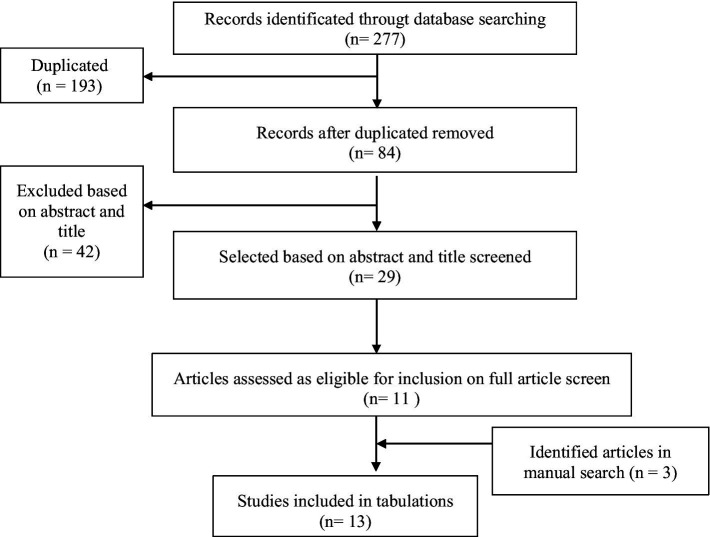
PRISMA flow chart.

Additionally, a complementary manual search was carried out by screening the reference lists of key studies and previous reviews. The study selection process is summarized in the PRISMA flow diagram (Figure 1), including identification, screening, eligibility, and inclusion stages.

Study titles were first screened to determine their relevance in addressing the research questions of this review. Based on this initial evaluation, a primary list of articles was compiled, followed by a second screening that included both titles and abstracts. Full-text versions of the selected articles were then retrieved and assessed for eligibility according to predefined inclusion criteria (see below). Studies that met these criteria underwent a detailed full-text review to determine their final inclusion.

#### 2.1.2 Inclusion and exclusion criteria

Studies were deemed eligible if they met the following criteria:

- Published between 2013 and 2023 in peer-reviewed scientific journals;
- Exclusive use of the selected instruments to assess both autistic traits (AQ or ADOS-2) and anxiety (HADS, STAI, GAD-7, BAI, or SCL-90-R);
- Inclusion of adult participants from the general population (aged 18 years or older), although one study including participants aged 15 and above was accepted due to the relevance of the data.

Studies were excluded if they met any of the following conditions:

- Case studies or single-case designs;
- Studies including only clinical populations with a formal ASD diagnosis or psychiatric comorbidities (e.g., anorexia nervosa);
- Studies measuring only one of the two variables (autism or anxiety).

#### 2.1.3 Selected studies

The initial search yielded 277 results. After removing duplicates, 84 articles remained for title and abstract screening. Forty-two studies were excluded during the initial screening for not meeting the inclusion criteria. The remaining 29 full-text articles were retrieved and assessed in detail. Disagreements between reviewers were resolved through discussion. Of these, 13 studies comprising 18 independent samples met all criteria for inclusion in the meta-analysis. Several studies reported more than one eligible independent sample, which were treated separately. The study selection process is visually summarized in the PRISMA flow diagram (Figure 1).

The main characteristics of the included studies—including instruments used, sample size, participant age, gender distribution, and outcome variables—are presented in Table 2. Studies that met general inclusion criteria but lacked the statistical data necessary for effect size calculation were included only in the systematic review but excluded from the meta-analysis.

**Table 2:** Main characteristics of the studies selected.

ID	Study	Autism test (Mean; SD or r)	Anxiety test (Mean, SD)	Targe group (age, range or M, SD)	*N*	Gender
1	Adachi et al., 2020	AQ-50 (20.6; 6.9)	STAI-R (46.2; 10.1)	US (18–27)	68	27 M/41 F
2	Aldridge et al., 2021	AQ-28 (64.77; 11.86)	HADS-A (8.97; 4.34)	Adults (23)	178	95 M/83 F
3	Barnett et al., 2021 – A	AQ-50 (18.19; 7.28)	HADS-A (7.97; 4.16)	US (17–70; 23.63; 101.4)	253	Males
4	Barnett et al., 2021 – B	AQ-50 (15.98; 7.44)	HADS-A (9.55; 4.36)	US (17–70; 23.63; 101.4)	407	Females
5	Cassidy et al., 2019	AQ-10 (*r* = 0.262)	GAD7 (*r* = 0.262)	US (18–67; 21.09; 6)	180	35 M/138 F/7 O
6	de Vries et al., 2019	AQ-10 (3.43; 2.10)	HADS-A (6.96; 3.99)	Adults (32.1; 13.4)	1,022	219 M/325 F
7	Galvin and Richards, 2023	AQ-50 (20.23; 7.61)	HADS-A (9.50; 4.59)	Adults (19–69; 33.13; 11.07)	228	114 M/114 F
8	Galvin et al., 2022 – A	AQ-50 (20.54; 7.58)	HADS-A (8.37; 4.20)	Adults (18–75; 28.65; 11.96)	653	Males
9	Galvin et al., 2022 – B	AQ-50 (18.97; 9.26)	HADS-A (10.19; 4.42)	Adults (18–75; 28.65; 11.96)	415	Females
10	Kerr-Gaffney et al., 2021	AQ-10 (2, 1.48)	HADS-A (7, 7)	Adults (22.16; 3.60)	67	Females
11	Kerr-Gaffney et al., 2020 – A	ADOS-2 (2.70; 2.52)	HADS-A (5.02; 3.09)	Adults (24.37; 4.43)	46	3 M/43 F
12	Kerr-Gaffney et al., 2020 – B	ADOS-2 (4.18; 4.46)	HADS-A (10.78; 5.07)	Adults (26.33; 8.04)	50	1 M/49 F
13	Richards et al., 2023 – A	AQ-50 (21.25; 8.02)	HADS-A (8.16; 4.49)	Adults (38.14; 13.13)	695	348 M/347 F
14	Richards et al., 2023 – B	AQ-50 (20.22; 7.43)	HADS-A (6.61; 4.79)	Adults (38.14; 13.13)	700	345 M/355 F
15	Schneider et al., 2016 – A	AQ-50 (15.13; 6.60)	BAI (50.13; 7.56)	Adults (29.71; 7.52)	49	Male
16	Schneider et al., 2016 – B	AQ-50 (11.95; 4.83)	BAI (49.16; 5.38)	Adults (29.53; 76)	19	Female
17	Toyomoto et al., 2022	AQ-J-10	GAD7	Adults (18–39)	845	394 M/451 F
18	Watanabe et al., 2021	AQ-j-21 (9.30; 3.83)	HADS (15.21; 6.53)	US (23.7; 1.67)	151	99 M/52 F

US: University students.

### 2.2 Data analysis

#### 2.2.1 Effect size calculation

Effect sizes were calculated based on the means and standard deviations reported in each study, using Fisher’s Z transformation. Standard errors of the effect sizes, along with their 95% confidence intervals and variance, were also calculated using the web-based effect size calculator, version 2023.11.27 (Wilson, 2023). When studies did not report effect sizes directly, they were calculated from means and standard deviations following established formulas (Borenstein et al., 2009). All statistical analyses were conducted using JAMOVI software, version 2.3.21.0.3.

A random-effects meta-analytic model was implemented to derive pooled effect size estimates, accounting for between-study variability. This model assumes that the effect sizes from different samples/studies may come from distinct populations, and that these populations have their own sampling distributions (Card, 2011a). Considering sample-level effects, our systematic analysis of potential moderating variables was performed by estimating random effects at the sample level. The diversity of experimental settings in individual studies (e.g., gender, educational level, and geographic region) justified the use of a random-effects model as an appropriate approach (Cooper, 2016).

#### 2.2.2 Moderator analyses

The *Q* test was applied to assess heterogeneity across the included studies. This test evaluates whether the observed variability in results exceeds what would be expected by chance alone. A significant *Q* statistic indicates that the studies likely do not share a common underlying effect, suggesting the presence of true variation between them

#### 2.2.3 Evaluation of publication bias

To evaluate potential publication bias, three complementary methods were employed. First, a funnel plot was generated to visually inspect the symmetry of effect sizes, as asymmetry may indicate the presence of bias (Card, 2011b). Second, the fail-safe N method was applied to estimate how many additional non-significant studies would be required to nullify the statistical significance of the overall effect size (Rosenthal, 1979). Third, Egger’s regression test was conducted to statistically assess funnel plot asymmetry, using linear regression to detect small-study effects that may reflect bias (Egger et al., 1997).

## 3 Results

### 3.1 Results of the systematic review

A total of 13 studies met the inclusion criteria, comprising 18 independent samples. The combined sample included 6,026 adult participants, with individual sample sizes ranging from 19 to 1,022. Most studies involved participants from the general population, although four explicitly targeted university students.

Regarding the assessment of autistic traits, 55% of the samples (*n* = 10) employed the full version of the Autism Spectrum Quotient (AQ-50), while 22% (*n* = 4) used the short-form AQ-10. Only two studies utilized the ADOS-2 as a clinical instrument.

Anxiety was most frequently assessed using the Hospital Anxiety and Depression Scale (HADS-A, *n* = 13), followed by the Beck Anxiety Inventory (BAI, *n* = 2), the Generalized Anxiety Disorder scale (GAD-7, *n* = 2), and the State–Trait Anxiety Inventory (STAI-R, *n* = 1).

Several studies reported separate analyses by sex, and were treated as distinct independent samples (e.g., Galvin et al., 2022; Schneider et al., 2016). For consistency in analysis, only the neurotypical group from Kerr-Gaffney et al. (2021) was retained.

A detailed summary of each study’s characteristics is presented in Table 2, which includes sample size, gender distribution, instruments used, and descriptive statistics. A broader descriptive summary of sample characteristics and instruments used across all included studies is provided in Table 3. As shown in Table 3, most studies used the AQ-50 and HADS-A as primary measurement tools, with balanced gender representation and predominantly large sample sizes. This provides a solid base for reliable effect estimation in the subsequent meta-analysis.

**Table 3:** Descriptive statistics of the studies included.

Variable	Identified categories	Counts (%)
Gender	Males	3 (17%)
	Females	4 (22.2%)
	Both	11 (61.1%)
Measure of autism	AQ-50	10 (55.6%)
	AQ-28	2 (11.1%)
	AQ-10	2 (11.1%)
	ADOS-2	2 (11.1%)
	AQ-J-10	1 (5.6%)
	AQ-J-21	1 (5.6%)
Measure of anxiety	STAI-R	1 (5.6%)
	HADS-A	13 (72.2%)
	GAD7	2 (11.1%)
	BAI	2 (11.1%)
Target group	University students	5 (28.7%)
	Normal adults	13 (72.2%)
Sample size	Small-sample studies	7 (38.9%)
	Large-sample studies	11 (61.1%)

### 3.2 Meta-analytic findings

The effect sizes and confidence intervals of the 18 independent samples included in the meta-analysis are summarized in Table 4, showing the correlation coefficients (r), standardized effect sizes (ES), standard errors (SE), and 95% confidence intervals (CI) for each sample.

**Table 4:** Main characteristics of the studies selected.

ID	*r*	ES	seES	95% CI
1	−0.8286	−1.1836	0.0867	−1.3535, −1.0136
2	0.9524	1.857	0.0532	1.7527, 1.9613
3	0.6532	0.7809	0.0447	0.6934, 0.8684
4	0.4675	0.5068	0.0353	0.4376, 0.5761
5	0.262	0.2683	0.0752	0.1209, 0.4156
6	−0.4843	−0.5286	0.0221	−0.572, −0.4852
7	0.6493	0.7741	0.047	0.682, 0.8662
8	0.7053	0.8779	0.0277	0.8236, 0.9321
9	0.5185	0.5743	0.0349	0.5059, 0.6428
10	−0.443	−0.476	0.0874	−0.6472, −0.3047
11	−0.3678	−0.3858	0.106	−0.5936, −0.1781
12	−0.5686	−0.6454	0.1015	−0.8444, −0.4464
13	0.7094	0.8861	0.0269	0.8334, 0.9387
14	0.7365	0.9427	0.0268	0.8902, 0.9951
15	−0.9267	−1.6346	0.1026	−1.8357, −1.4335
16	−0.9643	−2.0033	0.169	−2.3346, −1.672
17	0.15	0.1511	0.0345	0.0836, 0.2187
18	−0.4833	−0.5273	0.0578	−0.6406, −0.4139

Based on these 18 independent samples derived from the 13 selected studies, a meta-analysis was conducted to examine the relationship between autistic traits and anxiety in adult populations. The main statistical outcomes for each sample are provided in Table 4.

The overall mean effect size was 0.02, with a 95% confidence interval ranging from −0.44 to −0.48. According to Cohen’s (1988, 1992) guidelines, effect sizes of 0.20, 0.50, and 0.80 are considered small, medium, and large, respectively. Therefore, the findings indicate a positive but weak association between autistic traits and anxiety across the analyzed samples.

The *Q* statistic indicated significant heterogeneity (*Q* = 5222.91, df = 17, *p* < 0.001), suggesting that the variability in effect sizes exceeds what would be expected by chance. This justifies the use of a random-effects model, which accounts for both within- and between-study variability.

**Figure 2:**
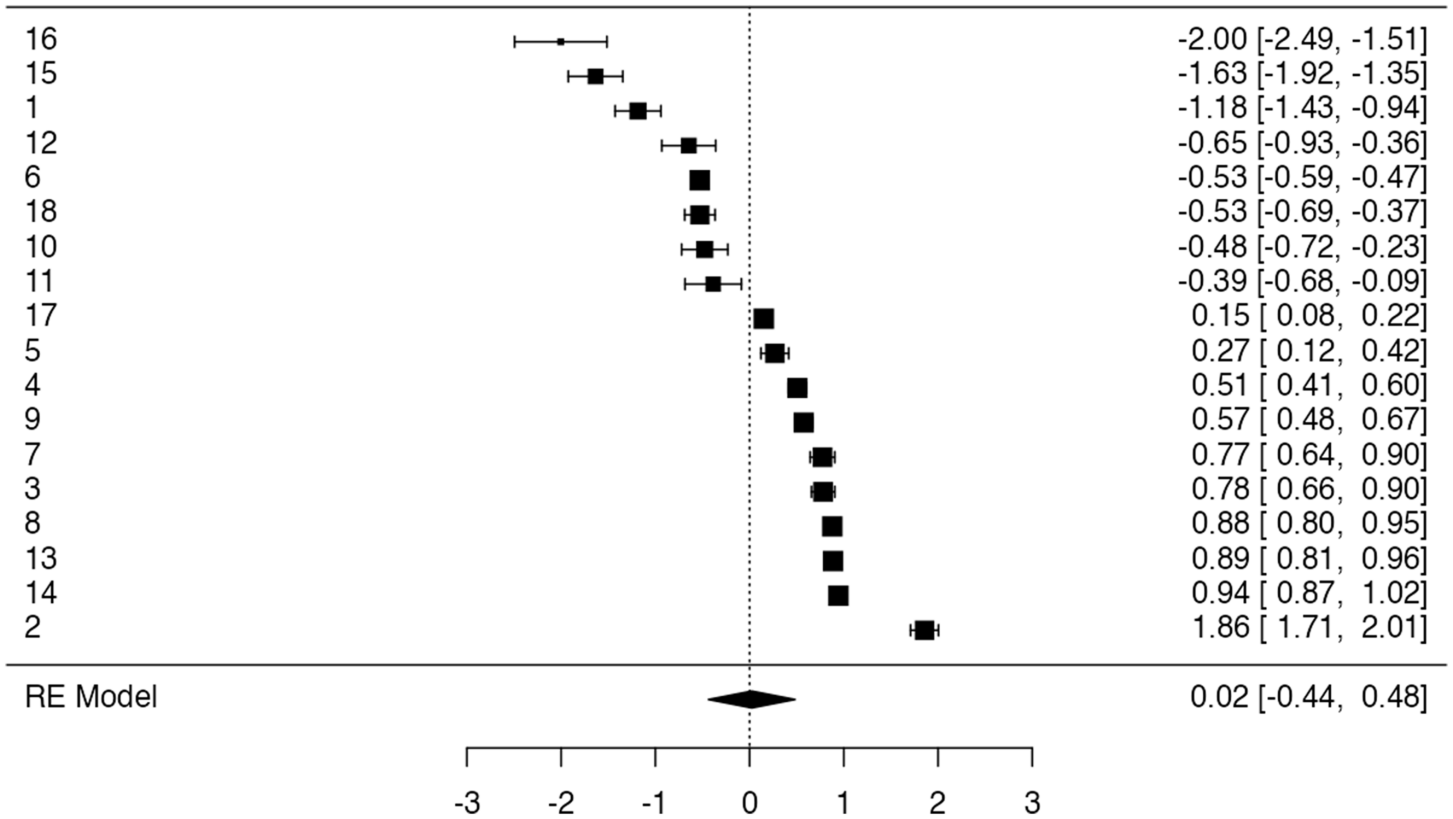
Forest plot main analysis.

The forest plot (Figure 2) visually displays the effect sizes and 95% confidence intervals for each of the 18 independent samples. Among them, ten reported positive effect sizes—specifically:

Toyomoto et al. (2022), Cassidy et al. (2019), Barnett et al. (2021) – B, Galvin et al. (2022) – B, Galvin and Richards (2023), Barnett et al. (2021) – A, Galvin et al. (2022) – A, Richards et al. (2023) – A, Richards et al. (2023) – B, and Aldridge et al. (2021). The remaining samples showed negative effect sizes.

Interestingly, most of the studies reporting positive associations had relatively large sample sizes, which corresponded with narrower confidence intervals and greater statistical precision. A notable exception was de Vries et al. (2019), which reported a negative effect size despite having the largest sample (*n* = 1,022); however, its narrow confidence interval reflects a high degree of reliability, regardless of the direction of the effect.

These results underscore the importance of sample size in shaping the interpretability and stability of effect size estimates across studies (Table 5).

**Table 5:** Relationship between autism and anxiety: overall results.

Model	*Q*	*k*	Weighted *r*	95% CI
Overall	5222.908	18	0.02	(−0.44–0.48)

### 3.3 Assessment of publication bias

**Figure 3:**
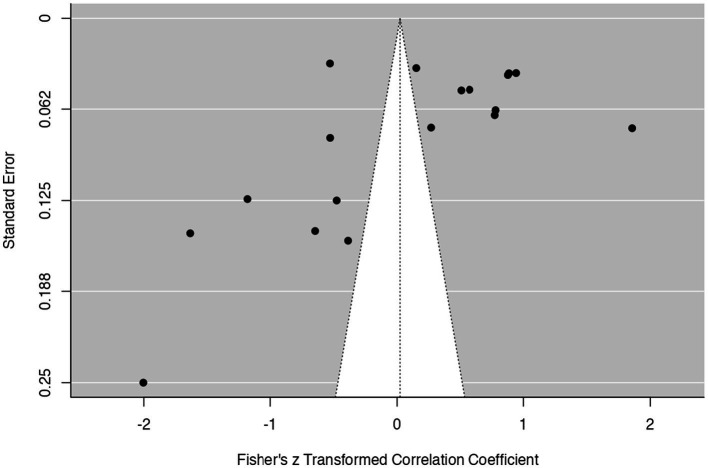
Funnel plot.

To evaluate potential publication bias, three complementary methods were applied: visual inspection of the funnel plot, Rosenthal’s fail-safe N calculation, and Egger’s regression test. The funnel plot (Figure 3) showed a noticeable asymmetry, with several studies deviating from the mean effect size estimate. This pattern suggests the possible absence of studies reporting small or non-significant effects, which may reflect publication bias.

Rosenthal’s fail-safe *N* test yielded a value of 5,085 (*p* < 0.001), indicating that over five thousand additional studies with null results would be needed to reduce the observed effect to non-significance. Although this high value suggests overall robustness, it does not eliminate concerns about selective reporting.

Kendall’s tau correlation was statistically significant and negative (*τ* = −0.373, *p* = 0.032), supporting the presence of asymmetry in the data distribution. Egger’s regression test further confirmed potential bias (*t* = −4.572, *p* < 0.001), highlighting the likelihood of systematic underrepresentation of studies with lower or non-significant effect sizes.

Taken together, these findings suggest that the meta-analytic results may be partially influenced by publication bias, and this should be considered when interpreting the magnitude of the overall effect.

## 4 Discussion

The present study aimed to systematically review the most recent empirical evidence on the relationship between autistic traits and anxiety symptoms in adults. Specifically, it sought to determine whether this association could be considered statistically significant based on studies published in the last decade that employed standardized and validated instruments to assess both constructs. This review focused on dimensional assessments of autistic traits rather than formal ASD diagnoses, aiming to provide clarity on the relationship between subclinical autism and anxiety, which is often conflated in the literature.

Findings from 13 articles (comprising 18 independent samples) revealed a mixed distribution: 55% of the studies reported positive effect sizes, whereas 45% yielded negative ones. However, the overall meta-analysis did not show a statistically significant association between autistic traits and anxiety (*g* = 0.0234, SE = 0.235, 95% CI = −0.438–0.483, *p* = 0.921). However, the direction and strength of the relationship appear to vary depending on the sample characteristics and measurement instruments used. The heterogeneity analysis yielded an I^2^ value of 99.83%, indicating considerable variability across studies. Graphically, studies with larger sample sizes tended to report positive associations, while studies with smaller samples more often showed negative effect sizes. Notably, the study by De Vries et al. (2019), which included the largest sample (*N* = 1,022), showed a negative effect size, but with a narrow confidence interval, suggesting high statistical precision rather than inconsistency.

It is worth highlighting that the data collected reflect the current reality regarding the considerable variability in how autistic traits are conceptualized and measured in adult populations. The high heterogeneity among the included studies is attributable not only to methodological differences but also to the absence of a unified framework for assessing autism in undiagnosed adults. Multiple versions of the Autism Spectrum Quotient (AQ)—such as the AQ-10, AQ-28, AQ-50, and AQ-j-21—were used inconsistently across studies, each with different lengths and psychometric properties, directly affecting comparability. This diversity also points to an ongoing debate within the research community regarding how to define and operationalize the broader autism phenotype (BAP). Moreover, most tools were originally designed for clinical populations, which may limit their sensitivity and validity when applied to subclinical contexts. This situation underscores the urgent need for the development and consensus on instruments specifically adapted to detect autistic traits in adults without formal diagnoses, as well as the promotion of studies that incorporate dimensional and contextual perspectives of autism. Consistent with our findings, other authors have also highlighted that variability in sample characteristics, diagnostic criteria, and assessment tools can lead to differing results across studies (Hikmiah, 2019). Moreover, the reliance on self-report anxiety measures (e.g., HADS, GAD-7), without clinical diagnosis confirmation, may limit the ability to distinguish between clinical and subclinical anxiety presentations.

From a theoretical standpoint, these findings suggest that while a statistically significant global association between subclinical autistic traits and anxiety was not confirmed, there is sufficient indication to warrant further investigation. A transdiagnostic approach appears particularly relevant, as it allows for the exploration of shared cognitive and affective mechanisms across both clinical and subclinical conditions. Mechanisms such as cognitive rigidity, goal-generation difficulties, or heightened preference for environmental consistency may underpin both autistic traits and anxiety.

Clinically, these findings underscore the need for more sensitive intervention models for adults who, although not meeting diagnostic criteria for Autism Spectrum Disorder (ASD), exhibit autistic-like traits alongside anxiety symptoms. Targeted interventions that address executive functioning, emotional self-regulation, and cognitive flexibility could be especially beneficial in reducing anxiety and improving quality of life in this population.

This study also presents several limitations. First, the high heterogeneity across studies may be related to the diverse versions of the AQ used, cultural differences, and varying methodological approaches. Second, the measurement tools employed may not be sufficiently sensitive to detect subclinical expressions of autistic traits. Third, anxiety was assessed exclusively through self-report measures, such as the HADS and GAD-7, which may not distinguish between clinical and subclinical anxiety presentations or capture the full complexity of the symptomatology. Lastly, dimensional variability in anxiety symptoms was not examined.

Future research should incorporate more specific instruments such as the Broad Autism Phenotype Questionnaire (BAPQ, Hurley et al., 2007), and aim to include formal clinical assessments for anxiety in addition to self-report tools. Furthermore, it would be valuable to explore potential moderators such as age, gender, cultural background, and the specific type of anxiety assessed. Mixed-methods designs and in-depth analyses of cognitive-affective mechanisms—such as generative social cognition and preference for environmental consistency—could contribute to a deeper understanding of the shared and unique features of autism and anxiety.

## Data Availability

The raw data supporting the conclusions of this article will be made available by the authors, without undue reservation.

## References

[ref1] Adachi Y. Yoshikawa H. Yokoyama S. Iwasa K. (2020). Characteristics of university students supported by counseling services: analysis of psychological tests and pulse rate variability. PLoS One 15:e0218357. doi: 10.1371/journal.pone.0218357, PMID: 32822354 PMC7446896

[ref2] Adams D. Clark M. Simpson K. (2020). The relationship between child anxiety and the quality of life of children, and parents of children, on the autism spectrum. J. Autism Dev. Disord. 50, 1756–1769. doi: 10.1007/s10803-019-03932-2, PMID: 30805767

[ref3] Aldridge Z. Patel S. Guo B. Nixon E. Pierre Bouman W. Witcomb G. L. . (2021). Long-term effect of gender-affirming hormone treatment on depression and anxiety symptoms in transgender people: a prospective cohort study. Andrology 9, 1808–1816. doi: 10.1111/andr.12884, PMID: 32777129

[ref4] Ambrose K. Simpson K. Adams D. (2022). The impact of anxiety on the participation of children on the autism spectrum. J. Autism Dev. Disord. 52, 2958–2969. doi: 10.1007/s10803-021-05162-x, PMID: 34196892

[ref5] American Psychiatric Association (2013). Diagnostic and statistical manual of mental disorders (DSM-5®): American Psychiatric Pub.

[ref6] Barnett A. Edwards K. Harper R. Evans E. Alexander D. Choudhary M. . (2021). The association between autistic traits and disordered eating is moderated by sex/gender and independent of anxiety and depression. J. Autism Dev. Disord. 51, 1866–1879. doi: 10.1007/s10803-020-04669-z, PMID: 32852639 PMC8124044

[ref7] Baron-Cohen S. Wheelwright S. Skinner R. Martin J. Clubley E. (2001). The autism-spectrum quotient (AQ): evidence from Asperger syndrome/high functioning autism, males and females, scientists and mathematicians. J. Autism Dev. Disord. 31, 5–17. doi: 10.1023/a:1005653411471, PMID: 11439754

[ref8] Barros C. Oliveira-Silva P. Alves N. T. Sampaio A. (2022). Autism traits dimensionality and multivariate relationship between autism traits, alexithymia, and trait anxiety in general population. BMC Psychol. *10*:221. doi: 10.1186/s40359-022-00923-3 36240538

[ref9] Beck A. T. Epstein N. Brown G. Steer R. (1988). An inventory for measuring clinical anxiety: Psychometric properties. J. Consult. Clin. Psychol. 56, 893–897. doi: 10.1037//0022-006x.56.6.893 3204199

[ref10] Borenstein M. Hedges L. V. Higgins J. P. Rothstein H. R. (2009). Introduction to meta-analysis: John Wiley & Sons, Ltd. doi: 10.1002/9780470743386

[ref11] Card N. A. (2011a). Applied meta-analysis for social science research. New York, NY: Guilford Press.

[ref12] Card N. A. (2011b). Metaanálisis aplicado a la investigación en ciencias sociales. Nueva York, NY: Guilford Press.

[ref13] Cassidy SA. Gould K. Townsend E. Pelton M. Robertson AE. Rodgers J. (2019). Is camouflaging autistic traits associated with suicidal thoughts and behaviours? Expanding the interpersonal psychological theory of suicide in an undergraduate student sample. J Autism Dev Disord. 50:3638–3648. doi: 10.1007/s10803-019-04323-3 PMC7502035 31820344

[ref14] Charlot L. Deutsch C. K. Albert A. Hunt A. Connor D. F. McIlvane W. J. Jr. (2008). Mood and anxiety symptoms in psychiatric inpatients with autism spectrum disorder and depression. J. Ment. Health Res. Intellect. Disabil. 1, 238–253. doi: 10.1080/19315860802313947, PMID: 24009649 PMC3760522

[ref9006] Cohen J. (1988). Statistical Power Analysis for the Behavioral Sciences. Hillsdale (NJ): Lawrence Erlbaum Associates Publishers.

[ref9007] Cohen J. (1992). Statistical Power Analysis. Current Directions in Psychological Science, 1. doi: 10.1111/1467-8721.ep10768783

[ref9001] Cooper A. F. (2016). The BRICS: a very short introduction. Oxford, UK: Oxford University Press, 144. doi: 10.1111/GOVE.12325

[ref15] Clark D. A. Beck A. T. (2012). The anxiety and worry workbook: The cognitive behavioral solution. New York, NY: The Guilford Press.

[ref16] De Vries A. L. Roehle R. Marshall L. Frisén L. Van De Grift T. C. Kreukels B. P. . (2019). Mental health of a large group of adults with disorders of sex development in six European countries. Psychosom. Med. 81, 629–640. doi: 10.1097/PSY.0000000000000718, PMID: 31232913 PMC6727927

[ref17] Demetriou E. A. Lampit A. Quintana D. S. Naismith S. L. Song Y. J. Pye J. E. . (2018). Autism spectrum disorders: a meta-analysis of executive function. Mol. Psychiatry 23, 1198–1204. doi: 10.1038/mp.2017.75, PMID: 28439105 PMC5984099

[ref18] Derogatis L. R. Lipman R. S. Covi L. (1977). SCL-90. Administration, scoring and procedures manual-I for the R (revised) version and other instruments of the psychopathology rating scales series. Chicago: Johns Hopkins University School of Medicine.

[ref19] Despert J. L. (1965). The emotionally disturbed child, then and now. New York: Vantage Press.

[ref9002] Egger M. Smith G. D. Schneider M. Minder C. (1997). Bias in meta-analysis detected by a simple, graphical test. bmj. 315, 629–634. doi: 10.1136/bmj.315.7109.629, PMID: 9310563 PMC2127453

[ref20] Galvin J. Evans E. H. Talbot C. V. Wilson C. Richards G. (2022). The associations between autistic traits and disordered eating/drive for muscularity are independent of anxiety and depression in females but not males. PLoS One 17:e0276249. doi: 10.1371/journal.pone.0276249, PMID: 36251679 PMC9576073

[ref21] Galvin J. Richards G. (2023). The indirect effect of self-compassion in the association between autistic traits and anxiety/depression: a cross-sectional study in autistic and non-autistic adults. Autism 27, 1256–1270. doi: 10.1177/13623613221132109 36341962

[ref22] Godoy-Gimenez M. Gonzalez-Rodriguez A. Cañadas F. Estévez A. F. Sayans-Jimenez P. (2018). Psychometric properties of the Spanish version of the broad autism phenotype questionnaire: strengths, weaknesses, and future improvements. J. Autism Dev. Disord. 48, 770–783. doi: 10.1007/s10803-017-3438-0, PMID: 29282584

[ref23] Hikmiah Z. (2019). Heterogeneity in anxiety disorder and autism spectrum disorder: difference and overlap. IJDS Indones. J. Disabil. Stud. 6, 269–281. doi: 10.21776/ub.IJDS.2019.006.02.18

[ref24] Hollocks M. J. Lerh J. W. Magiati I. Meiser-Stedman R. Brugha T. S. (2019). Anxiety and depression in adults with autism spectrum disorder: a systematic review and meta-analysis. Psychol. Med. 49, 559–572. doi: 10.1017/S0033291718002283, PMID: 30178724

[ref9003] Hollocks M. J. Ozsivadjian A. Matthews C. E. Howlin P. Simonoff E. (2013). The relationship between attentional bias and anxiety in children and adolescents with autism spectrum disorders. Autism Res. 6, 237–247., PMID: 23907924 10.1002/aur.1285

[ref25] Howlin P. (1998). Children with autism an Asperger syndrome. A guide for practitioners and cares. New York: John Wiley.

[ref26] Hurley R. S. Losh M. Parlier M. Reznick J. S. Piven J. (2007). The broad autism phenotype questionnaire. J. Autism Dev. Disord. 37, 1679–1690. doi: 10.1007/s10803-006-0299-3, PMID: 17146701

[ref27] Jenkinson R. Milne E. Thompson A. (2020). The relationship between intolerance of uncertainty and anxiety in autism: a systematic literature review and meta-analysis. Autism 24, 1933–1944. doi: 10.1177/1362361320932437, PMID: 32564625 PMC7539603

[ref28] Jobe L. E. White S. W. (2007). Loneliness, social relationships, and a broader autism phenotype in college students. Personal. Individ. Differ. 42, 1479–1489. doi: 10.1016/j.paid.2006.10.021

[ref29] Joshi G. Wozniak J. Petty C. Martelon M. K. Fried R. Bolfek A. . (2013). Psychiatric comorbidity and functioning in a clinically referred population of adults with autism spectrum disorders: a comparative study. J. Autism Dev. Disord. 43, 1314–1325. doi: 10.1007/s10803-012-1679-5, PMID: 23076506

[ref30] Kanner L. (1944). Early infantile autism. J. Pediatr. 25, 211–217.

[ref31] Kerr-Gaffney J. Harrison A. Tchanturia K. (2020). Autism spectrum disorder traits are associated with empathic abilities in adults with anorexia nervosa. J. Affect. Disord. 266, 273–281. doi: 10.1016/j.jad.2020.01.169, PMID: 32056888

[ref32] Kerr-Gaffney J. Hayward H. Jones E. J. Halls D. Murphy D. Tchanturia K. (2021). Autism symptoms in anorexia nervosa: a comparative study with females with autism spectrum disorder. Mol. Autism. 12:47. doi: 10.1186/s13229-021-00455-5, PMID: 34193255 PMC8247081

[ref33] Kurtz K. White L. C. Lutz M. (2023). The role of the broader autism phenotype in anxiety and mood disorders in young adults from different racial groups. Front. Psych. 14:1187298. doi: 10.3389/fpsyt.2023.1187298 PMC10278885 37342174

[ref34] Liberati A. Altman D. G. Tetzlaff J. Mulrow C. Gøtzsche P. C. Ioannidis J. P. . (2009). The PRISMA statement for reporting systematic reviews and meta-analyses of studies that evaluate health care interventions: explanation and elaboration. Ann. Intern. Med. 151:W65. doi: 10.1016/j.jclinepi.2009.06.006 19622512

[ref35] Lin L. Y. Huang P. C. (2019). Quality of life and its related factors for adults with autism spectrum disorder. Disabil. Rehabil. 41, 896–903. doi: 10.1080/09638288.2017.1414887, PMID: 29228834

[ref36] Lord C. Rutter M. DiLavore P. C. Risi S. Gotham K. Bishop S. L. (2012). Autism diagnostic observation schedule: Manual. Los Angeles: WPS.

[ref37] Lugo-Marin J. Magan-Maganto M. Rivero-Santana A. Cuellar-Pompa L. Alviani M. Jenaro-Rio C. . (2019). Prevalence of psychiatric disorders in adults with autism spectrum disorder: a systematic review and meta-analysis. Res. Autism Spectr. Disord. 59, 22–33. doi: 10.1016/j.rasd.2018.12.004

[ref38] Mazefsky C. A. Folstein S. E. Lainhart J. E. (2008). Overrepresentation of mood and anxiety disorders in adults with autism and their first-degree relatives: what does it mean? Autism Res. 1, 193–197. doi: 10.1002/aur.23, PMID: 19360666 PMC2939830

[ref39] Morrison K. E. Chambers L. K. Faso D. J. Sasson N. J. (2018). The content and function of interests in the broad autism phenotype. Res. Autism Spectr. Disord. 49, 25–33. doi: 10.1016/j.rasd.2018.02.002

[ref40] Ni W. Lu H. Wang Q. Song C. Yi L. (2023). Vigilance or avoidance: how do autistic traits and social anxiety in the general population separately and interactively affect attention to the eyes. Front. Neurosci. 16:1081769. doi: 10.3389/fnins.2022.1081769 36711128 PMC9876610

[ref41] Normansell-Mossa B. McNally A. Osbourne L. A. (2021). Sensory sensitivity and intolerance of uncertainty in autistic adults. Front. Psychol. 12:731753. doi: 10.3389/fpsyg.2021.731753 34867612 PMC8635111

[ref42] Page M. J. McKenzie J. E. Bossuyt P. M. Boutron I. Hoffmann T. C. Mulrow C. D. . (2021). The PRISMA 2020 statement: an updated guideline for reporting systematic reviews. BMJ 372:n71. doi: 10.1136/bmj.n71, PMID: 33782057 PMC8005924

[ref43] Park S. H. Song Y. J. C. Demetriou E. A. Pepper K. L. Norton A. Thomas E. E. . (2019). Disability, functioning, and quality of life among treatment-seeking young autistic adults and its relation to depression, anxiety, and stress. Autism 23, 1675–1686. doi: 10.1177/1362361318823925, PMID: 30654629

[ref44] Paula P. I. (2013). Coocurrencia entre ansiedad y autismo: Las hipótesis del error social y de la carga alostática. Revista de Neurologia 56, S45–S59. 23446724

[ref45] Paula I. (2016). La ansiedad en el autismo: Alianza Editorial.

[ref46] Richards G. Kelly S. Johnson D. Galvin J. (2023). Autistic traits and borderline personality disorder traits are positively correlated in UK and US adult men and women. Pers. Individ. Differ. 213:112287. doi: 10.1016/j.paid.2023.112287

[ref9004] Rosenthal R. (1979). The file drawer problem and tolerance for null result. Psychol. Bull. 86, 638.

[ref47] Ruzich E. Allison C. Smith P. Watson P. Auyeung B. Ring H. . (2015). Measuring autistic traits in the general population: a systematic review of the autism-Spectrum quotient (AQ) in a nonclinical population sample of 6,900 typical adult males and females. Mol. Autism. 6, 2–12. doi: 10.1186/2040-2392-6-2, PMID: 25874074 PMC4396128

[ref48] Sasson N. J. Nowlin R. B. Pinkham A. E. (2013). Social cognition, social skill, and the broad autism phenotype. Autism 17, 655–667. doi: 10.1177/1362361312455704, PMID: 22987889

[ref49] Schneider A. Johnston C. Tassone F. Sansone S. Hagerman R. J. Ferrer E. . (2016). Broad autism spectrum and obsessive–compulsive symptoms in adults with the fragile X premutation. Clin. Neuropsychol. 30, 929–943. doi: 10.1080/13854046.2016.1189536 27355445 PMC5004987

[ref50] Schopler E. Mesibov G. B. (Eds.) (1994). Behavioral issues in autism. New York: Plenum Press.

[ref51] Spielberger C. D. Gorsuch R. L. Lushene R. E. Cubero N. S. (1999). STAI: Cuestionario de ansiedad estado-rasgo. Madrid: TEA Ediciones.

[ref52] Spitzer RL. Kroenke K. Williams JB. (2006). Generalized anxiety disorder 7-item (GAD-7) scale. Arch Intern Med. 166:1092–1097. 16717171 10.1001/archinte.166.10.1092

[ref53] Townsend A. N. Guzick A. G. Hertz A. G. Kerns C. M. Goodman W. K. Berry L. N. . (2022). Anger outbursts in youth with ASD and anxiety: phenomenology and relationship with family accommodation. Child Psychiatry Hum. Dev. 55, 1259–1268. doi: 10.1007/s10578-022-01489-3, PMID: 36576640 PMC10300226

[ref54] Toyomoto R. Sakata M. Yoshida K. Luo Y. Nakagami Y. Iwami T. . (2022). Validation of the Japanese big five scale short form in a university student sample. Front. Psychol. 13:862646. doi: 10.3389/fpsyg.2022.862646, PMID: 35814124 PMC9262100

[ref55] Tsakanikos E. Underwood L. Kravariti E. Bouras N. McCarthy J. (2011). Gender differences in co-morbid psychopathology and clinical management in adults with autism spectrum disorders. Res. Autism Spectr. Disord. 5, 803–808. doi: 10.1016/j.rasd.2010.09.009

[ref9005] Van Steensel F. J. Bögels S. M. Perrin S. (2011). Anxiety disorders in children and adolescents with autistic spectrum disorders: A meta-analysis. Clin. Child Fam. Psychol. Rev. 14, 302–317., PMID: 21735077 10.1007/s10567-011-0097-0 PMC3162631

[ref56] Velikonja T. Fett A. K. Velthorst E. (2019). Patterns of nonsocial and social cognitive functioning in adults with autism spectrum disorder: a systematic review and meta-analysis. JAMA Psychiatr. 76, 135–151. doi: 10.1001/jamapsychiatry.2018.3645 PMC6439743 30601878

[ref57] Watanabe T. Kondo M. Sakai M. Takabatake S. Furukawa T. A. Akechi T. (2021). Association of autism spectrum disorder and attention deficit hyperactivity disorder traits with depression and empathy among medical students. Adv. Med. Educ. Pract. 12, 1259–1265. doi: 10.2147/AMEP.S334155 34737666 PMC8560068

[ref58] Wheelwright S. Auyeung B. Allison C. Baron-Cohen S. (2010). Defining the broader, medium and narrow autism phenotype among parents using the autism Spectrum quotient (AQ). Mol. Autism. 1, 1–9. doi: 10.1186/2040-2392-1-10 20678260 PMC2913943

[ref59] Wilson D. B. (2023). Practical meta-analysis effect size calculator (Version 2023.11.27). Avilable online at: https://www.campbellcollaboration.org/escalc/html/

[ref60] Wing L. Gould J. Yeates S. R. Brierly L. M. (1977). Symbolic play in severely mentally retarded and in autistic children. J. Child Psychol. Psychiatry 18, 167–178. doi: 10.1111/j.1469-7610.1977.tb00426.x 141458

[ref61] Wood J. J. Gadow K. D. (2010). Exploring the nature and function of anxiety in youth with autism spectrum disorders. Clin. Psychol. Sci. Pract. 17:281. doi: 10.1111/j.1468-2850.2010.01220.x

[ref62] Zigmond A. S. Snaith R. P. Kitamura T. (1993). The hospital anxiety and depression scale (HAD). Arch. Psychiatr. Diagnost. Clin. Eval. 4, 371–372. doi: 10.1111/j.n1983.tb09716.x

